# Rethinking barriers and facilitators in implementation science: conceptual, methodological, and practical challenges, and ways forward

**DOI:** 10.1186/s43058-026-00901-6

**Published:** 2026-03-18

**Authors:** Per Nilsen

**Affiliations:** 1 Department of Health, Medicine and Health Sciences, Linköping University, Linköping, Sweden; 2 School of Health and Welfare, Halmstad University, Halmstad, Sweden

**Keywords:** Barriers, Facilitators, Determinant frameworks, Mechanisms, Context, Implementation strategies, Theoretical development

## Abstract

Implementation science has long relied on the concepts of barriers and facilitators (B&F) to explain why evidence-based practices succeed or fail. Determinant frameworks such as the Consolidated Framework for Implementation Research and Theoretical Domains Framework have standardized this terminology, offering a common vocabulary to describe influences on implementation processes. However, the increasing maturity of the field exposes conceptual and methodological limitations of the traditional B&F approach. This article critically examines eight interrelated challenges in the identification, measurement, and application of B&F, drawing on theoretical, empirical, and methodological literature.

The analysis highlights that B&F are not simple opposites, that they are often based on perceptions rather than mechanisms, and that exhaustive identification may not be feasible or conceptually coherent. Further, measurement and prioritization remain inconsistent, and the dynamic, multi-level, and temporal dimensions of implementation are frequently overlooked. The persistent gap between identifying B&F and linking them to implementation strategies constrains progress toward explanatory and actionable science.

Moving beyond descriptive checklists, the paper suggests reconceptualizing B&F as dynamic system properties and adopting methods that capture complexity, mechanisms, and contextual interaction. Strengthening theory–method alignment will enable implementation research to evolve from enumerating determinants toward explaining how change unfolds in real-world systems.

Contributions to the literature • Highlights conceptual and methodological limitations of the dominant barriers-and-facilitators paradigm.

• Introduces eight critical challenges that restrict explanatory progress in implementation science.

• Proposes a reconceptualization of barriers and facilitators as dynamic system properties rather than static determinants.

• Advocates for causal, configurational, and complexity-informed approaches to understand mechanisms of change.

• Offers guidance for linking determinants, mechanisms, and implementation strategies.

## Introduction

Implementation science seeks to understand how evidence-based practices (EBPs) can be integrated effectively into real-world settings. Central to this endeavor is identifying the factors that influence implementation success or failure. The concepts of barriers and facilitators (B&F) have become foundational in this field, offering a structured lens through which challenges, design strategies, and outcomes can be evaluated. Determinant frameworks, such as the Consolidated Framework for Implementation Research (CFIR) [1] and the Theoretical Domains Framework (TDF) [2], have further standardized how these factors are conceptualized, categorized, and reported, thereby enhancing conceptual clarity and enabling greater consistency and comparability across studies [3].

However, as implementation science matures, there is increasing recognition that the traditional B&F approach may not fully capture the complexity of real-world implementation processes [4–6]. Implementation involves dynamic interactions among individuals, organizations, and broader systems, which may not be adequately represented through lists of determinants [7–10]. The continued reliance on determinant frameworks shapes how data are collected, interpreted, and translated into strategy development, potentially constraining theoretical advancement and practical innovation.

This study is motivated by the need to critically examine the conceptual foundations and methodological implications of the B&F approach. By exploring its assumptions and limitations, the study aims to clarify how the field can more effectively conceptualize and study the factors that shape implementation. Such reflection is essential for refining the theoretical tools that underpin implementation science research and for advancing more explanatory and dynamic understandings of change in complex systems.

An integrative, theory-informed review of the literature was conducted to identify and analyze key conceptual and methodological challenges in the current use of B&F within implementation science. The analysis is informed by a broadly critical realist orientation, assuming that implementation processes are shaped by real but often unobservable structures, mechanisms, and contextual conditions that generate observed outcomes [11]. From this perspective, B&F are treated not as static or discrete factors, but as surface-level descriptions of deeper causal mechanisms operating within complex systems. At the same time, the paper is guided by a pragmatic stance consistent with much of implementation science, focusing on the practical consequences of prevailing analytic conventions and on how alternative ways of conceptualising determinants may better support explanation, learning, and action in real-world settings [6].

Foundational determinant frameworks such as the CFIR and TDF were examined alongside empirical and conceptual papers addressing the identification, measurement, and use of B&F in implementation science studies. Determinant frameworks were the primary focus because they are among the most widely used and cited approaches in implementation science for identifying and organising B&F. As such, they represent a central and influential way in which the barriers-and-facilitators logic is operationalised in the field.

This work is also informed by nearly 20 years of research in implementation science, which has generated extensive experience and critical reflection relevant to the issues examined in this paper. The analysis was iterative and interpretive rather than exhaustive, focusing on conceptual clarity, methodological limitations, and theoretical implications. Through this process, eight recurrent and interrelated challenges were identified, each reflecting a distinct tension in how B&F are conceptualized, operationalized, and applied across studies. The references cited are intended to be illustrative rather than exhaustive, drawing on a broader body of conceptual, methodological, and empirical literature reviewed for this paper. Well-recognised and representative examples were selected to demonstrate recurrent patterns and tensions in the use of B&F, rather than to provide a systematic or comprehensive review of the field.

## Findings

This section presents eight conceptual and methodological challenges in the current use of B&F within implementation science. For each of the eight issues, the paper outlines the central challenge, illustrate its implications for research and practice, and propose potential directions for conceptual and methodological refinement.

### Are facilitators the opposite of barriers?

#### Challenges

Determinant frameworks in implementation science typically assume that facilitators are mirror images of barriers, i.e., points on a single continuum ranging from hindrance to support. From this perspective, reducing barriers automatically increases facilitation, and vice versa [1, 12]. However, this assumption oversimplifies the dynamics of change and risks obscuring important heterogeneity within implementing settings. For example, implementers may experience the same structural condition (e.g. leadership support or workload) very differently, depending on professional role, motivation or prior experience.

Moreover, not all determinants plausibly lie on a single continuum. Some facilitators (e.g., intrinsic motivation, a sense of professional identity, shared purpose) may have no direct barrier equivalent, and conversely, the removal of a barrier (e.g., inadequate staffing or lack of resources) does not necessarily produce positive results. At the same time, certain determinants, such as physical space or access to equipment, may reasonably be conceptualised as continua, where increases or decreases directly affect implementation capacity. Treating all determinants as mirror-image barriers and facilitators therefore risks analytical imprecision.

Herzberg’s Two-Factor Theory of Motivation [13] provides a valuable conceptual framework for understanding this asymmetry. Herzberg distinguished between hygiene factors (extrinsic conditions such as pay, supervision, and work environment) and motivators (intrinsic factors such as achievement, recognition, and personal growth). The absence of hygiene factors produces dissatisfaction, but their presence does not generate satisfaction –only the absence of discontent. In contrast, motivators actively promote satisfaction and engagement, but their absence does not necessarily produce dissatisfaction.

#### Way forward

Future implementation science research should explicitly distinguish between barrier-type (hygiene) and facilitator-type (motivator) determinants, rather than assuming they exist on a single continuum. This conceptual refinement can guide the design of complementary strategies: barrier-reduction strategies should focus on ensuring basic enabling conditions, and facilitator-enhancement strategies should cultivate factors such as intrinsic motivation and shared ownership of change. By embracing Herzberg’s distinction [13], implementation science can move beyond removing obstacles toward actively building the motivational and relational foundations that sustain meaningful change.

### Are the B&F real or perceived?

#### Challenges

Studies of implementation determinants often rely on participants’ perceptions [14, 15], which may not always align with the actual mechanisms influencing implementation outcomes. In many cases, data are gathered through self-reporting methods such as interviews, focus groups, or surveys that, if used or interpreted uncritically, capture how individuals believe certain factors act as B&F, rather than how these factors actually operate in practice [16, 17]. This introduces a risk of conflating perceived and experienced determinants. Nevertheless, perceived determinants should not be treated as analytically trivial. Even when they do not directly correspond to underlying causal mechanisms, stakeholder perceptions may shape engagement, trust, and willingness to act, and can therefore influence implementation in meaningful ways.

Perceptions are shaped by context, professional identity, expectations, and social desirability [18, 19], which can distort how determinants of change are represented. Staff may cite lack of time or funding as barriers because these are socially acceptable explanations, even when deeper issues, such as unclear roles or low psychological safety, are more influential. Similarly, facilitators such as improved skills, stronger leadership, or additional resources are often proposed but may remain hypothetical rather than empirically validated. The key challenge is to distinguish determinants that are experienced and actionable from those that are merely perceived or aspirational; otherwise, strategies risk targeting surface perceptions instead of the structural or behavioral mechanisms that truly drive change. At the same time, failing to engage with perceived barriers that are salient to implementers may undermine trust and buy-in, even when those perceptions do not fully explain observed outcomes.

#### Way forward

Implementation science research should systematically differentiate between perceived and actual B&F to advance conceptual and empirical clarity. Mixed-method approaches can be particularly useful: perceptual data (e.g., qualitative insights about what stakeholders believe hinders or helps implementation) can be triangulated with objective indicators (e.g., audit data, workflow analysis) to validate whether perceived determinants translate into measurable effects. However, addressing perception–reality mismatches does not require invalidating stakeholder perspectives. Instead, perceptions can be treated as an entry point for dialogue and sense-making, helping to surface underlying concerns, relational dynamics, or structural constraints.

Implementation science studies might incorporate a two-dimensional classification of determinants, mapping both their ontological status (real versus perceived) and their influence level (proximal versus distal). Such mapping would help identify which determinants warrant strategies and which are better understood as contextual narratives. In some cases, strategies aimed at engaging with or reshaping perceptions, such as shared reflection, feedback, or co-interpretation of data, may be a necessary precursor to addressing more distal determinants and can help build trust and buy-in for subsequent change efforts.

### Can we identify all B&F?

#### Challenges

A key challenge of implementation science lies in the assumption, often implicit, that researchers can comprehensively identify all B&F relevant to a given implementation effort [17]. Determinant frameworks such as CFIR [1] and TDF [2] contain dozens of constructs that aim to capture the breadth of potential influences, from individual knowledge to organizational climate to external policies. Yet implementation contexts are complex adaptive systems, characterized by dynamic interdependencies, feedback loops, and emergent properties [7]. In such systems, it is neither feasible nor conceptually coherent to aim for complete identification of B&F. This challenge is particularly salient for societal- and systems-level determinants, including historical, structural, and policy forces that shape implementation contexts but may not be directly modifiable within a single initiative.

Comprehensiveness also risks conflating breadth with depth. Efforts to list every possible determinant may yield a superficial understanding without clarifying which mechanisms are at work. Conversely, focusing narrowly on context-specific B&F can generate rich but non-generalizable insights [10]. A narrow focus on readily observable or seemingly actionable determinants may also obscure how broader structural conditions influence more proximal factors, such as beliefs, expectations, or professional norms.

#### Way forward

Researchers should aim for theoretical sufficiency rather than exhaustive identification. This means defining clear inquiry boundaries, guided by theory, logic models, or program theory, and focusing on determinants plausibly linked to mechanisms of change [20]. Importantly, this does not imply privileging inner-setting or immediately modifiable determinants; outer-setting, historical, and structural influences warrant analytic attention even when they fall outside the locus of direct intervention.

Systems mapping [21, 22] and realist evaluation [23] help reveal not only which determinants exist, but how they interact in context to produce outcomes, including how distal societal or policy-level forces shape more proximal mechanisms such as trust, motivation, or attitudes. Research shows the limits of trying to identify every barrier; qualitative studies often capture B&F more effectively than surveys, which depend on pre-specified lists and assume prior knowledge of all relevant determinants [17, 24, 25]. This underscores the importance of theoretical sufficiency and contextual sensitivity over completeness, emphasizing the identification of key mechanisms driving change while remaining attentive to equity-relevant structural conditions that shape implementation possibilities.

### Can we measure the B&F we identify?

#### Challenges

Even when relevant B&F are identified, their measurement poses substantial challenges. Many constructs, such as organizational readiness or leadership engagement, are complex, context-dependent, and multi-dimensional [26, 27]. Quantitative measures often lack psychometric validation [28, 29], and qualitative assessments, although rich, can be difficult to standardize or compare [30].

Measurement difficulties also arise from the overlapping nature of constructs across frameworks, leading to redundancy and conceptual ambiguity. For example, culture, climate, and norms often appear under different labels but describe similar underlying phenomena [14]. Without clear operational definitions and validated tools, comparing findings across studies becomes problematic.

#### Way forward

To advance measurement, the field should embrace mixed-method and pragmatic measurement principles [28]. Quantitative scales should be developed and validated for reliability, sensitivity, and contextual fit, and qualitative methods can capture emergent or unanticipated determinants. Iterative, longitudinal measurement, such as repeated surveys [31] or ethnographic observation [32], can track the evolution of B&F over time. Measurement frameworks such as the Implementation Outcomes Framework [33] and the Psychometric and Pragmatic Evidence Rating Scale (PAPERS) [34] can guide the development of robust instruments.

### Can we determine which B&F are most important?

#### Challenges

Not all determinants exert equal influence on implementation outcomes. Yet in practice, importance is often inferred from the frequency of mentions in qualitative data or from the magnitude of association in quantitative models [35, 36]. These approaches risk conflating prevalence with causal importance. Some determinants may be necessary for success (e.g., leadership support), whereas others are important but not sufficient (e.g., positive attitudes). Distinguishing among these categories requires moving beyond descriptive methods toward causal inference.

Assessing importance is further complicated by the interdependence and context-specific nature of determinants. A factor that is highly influential in one setting may have limited relevance in another, depending on the organizational culture, resource availability, or phase of implementation [8]. Moreover, determinants rarely operate in isolation; their apparent importance may reflect interactions with other factors rather than intrinsic strength [14]. Attempts to rank B&F by frequency or statistical weight risk oversimplifying these contextual dynamics and ignoring mechanisms of action [10]. Without explicit theoretical grounding, such rankings may favor easily measurable determinants over those that are less visible but more causally significant.

#### Way forward

Causal and configurational approaches offer promising avenues. Necessary Condition Analysis (NCA) can test whether the absence of a determinant prevents success [37], and Qualitative Comparative Analysis (QCA) identifies combinations of determinants sufficient for desired outcomes [38]. Such methods allow researchers to explore not only which factors matter, but in what configurations and under what conditions. Mechanistic reasoning by examining the processes through which B&F exert influence can further clarify the importance, shifting focus from correlation to causation [10, 39].

### Can we determine which combinations of B&F are most important?

#### Challenges

Implementation success rarely depends on a single B&F acting in isolation. Instead, outcomes arise from configurations of interacting factors across multiple levels: individual, organizational, and system [8]. Despite this, most studies analyze B&F individually [3], using variable-centered methods such as regression, which assume additive and independent effects [40].

By neglecting interactions, the field risks mischaracterizing the causal structure of implementation processes. For instance, strong leadership may compensate for limited resources, and the same resources may be insufficient without a supportive culture. Understanding these interactions is essential for designing tailored implementation strategies. Seemingly minor barriers can interact synergistically to create substantial obstacles [4, 14]. The interplay among individual, organizational, and systemic determinants reveals that isolated analysis of single factors obscures the emergent and often unpredictable dynamics that characterize real-world practice change [10].

#### Way forward

Configurational and systems-based approaches can capture these complex interdependencies. QCA [38, 41], network analysis [42], and agent-based modeling [43] allow exploration of how combinations of determinants jointly shape outcomes. Longitudinal case studies [44, 45] and cross-case synthesis [46] can reveal recurring configurations across contexts. Adopting a complexity lens, recognizing nonlinearity, emergence, and adaptation, can help interpret why similar B&F sometimes yield divergent results across settings [4].

### Can we capture B&F over time, across levels, and among professional groups?

#### Challenges

Implementation processes unfold over time, yet most B&F studies are cross-sectional [1, 47]. Determinants may emerge, intensify, or dissipate as EBPs progress through exploration, adoption, and sustainment phases [48]. Similarly, different professional groups (e.g., clinicians, managers, policymakers) may experience distinct sets of B&F depending on role, power, and perspective [49]. Multi-level dynamics, such as the interaction between organizational culture and individual motivation, further complicate the picture [1].

Static, one-time assessments risk missing the temporal and relational evolution of determinants. They also obscure cross-level feedback; for example, early individual enthusiasm may trigger organizational investment, which in turn sustains engagement. Moreover, implementation science research frequently favors individual-level determinants, such as practitioner knowledge or motivation, and underemphasizes organizational and systemic influences [50]. This imbalance limits understanding of how multi-level interactions shape outcomes over time and across professional groups.

#### Way forward

Researchers should use longitudinal and multi-level designs that capture how B&F change over time and across groups [48, 51]. Realist-informed longitudinal case studies can trace how determinants emerge, interact, and transform [23, 52]. Methods such as time-series analysis [53], repeated-measures surveys [31], and participatory inquiry [54, 55] can provide temporal depth. Attention to professional subcultures and positionality ensures that multiple perspectives are represented, revealing how different actors perceive and influence implementation determinants [49].

### Can B&F be matched with relevant strategies?

#### Challenges

Identifying B&F is valuable only to the extent that it informs implementation strategies. Yet many studies stop at identification, offering little guidance on how to select or tailor strategies to address specific determinants. The link between determinants and strategies remains one of the field’s weakest areas [56]. Without a clear, evidence-based rationale connecting what hinders or facilitates implementation to the strategies intended to modify those conditions, efforts risk becoming ad hoc and inefficient.

Taxonomies such as the Expert Recommendations for Implementing Change (ERIC) [57] provide extensive lists of potential strategies, and recent efforts have sought to map ERIC strategies to CFIR constructs [58]. However, such mapping is often descriptive rather than mechanistic; it identifies potential matches but rarely explains the causal logic linking determinants, mechanisms, and outcomes. As a result, strategy selection can become a checklist exercise rather than a theory-driven process of change. Without specifying why or how a particular strategy should address a given barrier, researchers risk misalignment between the problem diagnosed and the solution applied. Strengthening this linkage is essential for building cumulative knowledge about which strategies work, under what conditions, and through which mechanisms.

#### Way forward

Advancement requires determinant–mechanism–strategy logic models that explicitly link B&F to hypothesized mechanisms of change and to corresponding strategies [10, 36]. For example, if lack of knowledge is identified as a barrier, educational strategies may be warranted, but only if knowledge deficits are truly the limiting mechanism. Using causal pathway modeling [36, 59] or realist logic models [60], researchers can test whether addressing a particular barrier (or leveraging a facilitator) leads to measurable improvement.

## Discussion

The eight challenges identified in this analysis share a common concern: the limitations of treating B&F as static, independent variables. Although the B&F paradigm has provided a valuable structure for identifying factors that influence implementation, it now risks constraining theoretical and methodological advancement. Implementation is a dynamic process shaped by interactions among people, contexts, and systems. Thus, frameworks and methods that favor categorization over explanation capture only a partial view of how change occurs.

The field would benefit from shifting from descriptive enumeration toward explanatory understanding. B&F should be conceptualized not as fixed attributes to be identified and tallied but as emergent system properties, patterns of interaction that evolve over time. From this perspective, B&F are dynamic features of implementation systems rather than discrete entities to be removed or enhanced. This reconceptualization invites greater attention to mechanisms; i.e., the processes through which determinants influence outcomes.

Methodologically, this shift calls for approaches capable of capturing complexity, interaction, and change over time. Realist evaluation, complexity-informed design, and systems mapping exemplify methodologies that can trace how determinants interact across multiple levels and contexts. Another crucial step is to integrate theory-driven prioritization and strategy linkage into research practice. Too often, studies identify a broad range of determinants without applying clear criteria for their relative importance or providing actionable guidance for strategy design. Theory-driven prioritization involves conceptual models or logic frameworks to determine which determinants are most likely to influence key mechanisms of change. Strategy linkage, in turn, connects these prioritized determinants to implementation strategies that directly target the mechanisms they enable or constrain. Embedding this logic within both research and practice strengthens the bridge between diagnostic insight and effective action.

Moving beyond the traditional B&F paradigm also requires a cultural shift within implementation science. Researchers, reviewers, and funders need to value methodological pluralism and theoretical innovation alongside standard determinant mapping. Embracing complexity means accepting that not all determinants can, or should, be isolated or ranked. Instead, progress lies in developing mid-range theories and methodological tools that can explain how configurations of factors interact and adapt within specific contexts.

In summary, the determinant/B&F framework remains foundational to implementation science, but it requires conceptual renewal to remain fit for purpose. By reconceptualizing B&F as dynamic system properties, adopting explanatory and complexity-informed methods, integrating theory-based prioritization and strategy linkage, and embedding equity perspectives, the field can evolve toward a more robust and actionable understanding of implementation. This shift, from enumeration to explanation, and from static factors to dynamic mechanisms, offers a pathway toward a more mature and impactful implementation science.

## Conclusions

B&F remain indispensable concepts in implementation science, but their current use requires critical refinement. The challenges outlined here, ranging from identification and measurement to causal prioritization, combinatorial complexity, and strategy alignment, underscore the need for a more dynamic, theory-driven, and action-oriented approach.

To move forward, implementation science must embrace theoretical sufficiency rather than exhaustive identification of determinants; advance rigorous and pragmatic measurement strategies; use causal and configurational methods to determine importance and combinations; treat B&F as distinct constructs with potentially asymmetric effects; capture temporal and multi-level dynamics; and integrate determinants into mechanism-based strategy selection frameworks. By evolving beyond descriptive checklists toward explanatory and strategic models, the field can better harness the conceptual power of B&F to inform effective, equitable, and sustainable implementation.

## Data Availability

Not applicable.

## References

[1] Damschroder LJ, Aron DC, Keith RE, Kirsh SR, Alexander JA, Lowery JC. Fostering implementation of health services research findings into practice: a consolidated framework for advancing implementation science. Implement Sci. 2009;4:50. 10.1186/1748-5908-4-50. 19664226 10.1186/1748-5908-4-50 PMC2736161

[2] Michie S, Johnston M, Abraham C, Lawton R, Parker D, Walker A. Making psychological theory useful for implementing evidence-based practice: the Theoretical Domains Framework. Implement Sci. 2005;1(1):29. 10.1186/1748-5908-2-29. 10.1136/qshc.2004.011155 PMC1743963 15692000

[3] Nilsen P. Making sense of implementation theories, models and frameworks. Implement Sci. 2015;10(1):53. 10.1186/s13012-015-0242-0. 25895742 10.1186/s13012-015-0242-0 PMC4406164

[4] Braithwaite J, Churruca K, Long JC, Ellis LA, Herkes J. When complexity science meets implementation science: a theoretical and empirical analysis of systems change. BMC Med. 2018;16(1):63. 10.1186/s12916-018-1057-z. 29706132 10.1186/s12916-018-1057-z PMC5925847

[5] Nilsen P, Birken S. Prologue. In: Nilsen P, Birken S editors, Handbook on implementation science. Cheltenham: Elgar Publishing; 2020. p. 1–7. 10.4337/9781788975995.00006

[6] Greenhalgh T, Papoutsi C. Studying complexity in health services research: desperately seeking an overdue paradigm shift. BMC Med. 2018;16(1):95. 10.1186/s12916-018-1089-4. 29921272 10.1186/s12916-018-1089-4 PMC6009054

[7] Hawe P, Shiell A, Riley T. Theorising interventions as events in systems. Am J Community Psychol. 2019;64(3–4):378–87. 10.1002/ajcp.12334. 10.1007/s10464-009-9229-9 19390961

[8] Pfadenhauer LM, Gerhardus A, Mozygemba K, Lysdahl KB, Booth A, Hofmann B, et al. Making sense of complexity in context and implementation: the Context and Implementation of Complex Interventions (CICI) framework. Implement Sci. 2017;12(1):21. 10.1186/s13012-017-0552-5. 28202031 10.1186/s13012-017-0552-5 PMC5312531

[9] Skivington K, Matthews L, Simpson SA, Craig P, Baird J, Blazeby JM, et al. A new framework for developing and evaluating complex interventions: update of Medical Research Council guidance. BMJ. 2021;374:n2061. 10.1136/bmj.n2061. 34593508 10.1136/bmj.n2061 PMC8482308

[10] Geng EH, Powell B, Goss CW, Lewis CC, Sales AE, Kim B. When the parts are greater than the whole: how understanding mechanisms can advance implementation research. Implement Sci. 2025;20(1):22. 10.1186/s13012-025-01427-6. 40361178 10.1186/s13012-025-01427-6 PMC12070568

[11] Bhaskar R. A realist theory of science. London: Routledge; 1978.

[12] Fleuren M, Wiefferink K, Paulussen T. Determinants of innovation within health care organizations: literature review and Delphi study. Int J Qual Health Care. 2004;16(2):107–23. 10.1093/intqhc/mzh030. 15051705 10.1093/intqhc/mzh030

[13] Herzberg F. The motivation to work. 2nd ed. New York: John Wiley; 1959.

[14] Nilsen P, Bernhardsson S. Context matters in implementation science: a scoping review of determinant frameworks that describe contextual determinants for implementation outcomes. BMC Health Serv Res. 2019;19:189. 10.1186/s12913-019-4015-3. 30909897 10.1186/s12913-019-4015-3 PMC6432749

[15] Birken SA, Powell BJ, Shea CM, Haines ER, Kirk MA, Leeman J, et al. Criteria for selecting implementation science theories and frameworks: results from an international survey. Implement Sci. 2017;12(1):124. 10.1186/s13012-017-0656-y. 29084566 10.1186/s13012-017-0656-y PMC5663064

[16] Harvey G, Kitson A. Implementing evidence-based practice in healthcare: a facilitation guide. Abingdon, UK: Routledge; 2016. 10.4324/9780203557334.

[17] Nilsen P, Bernhardsson S. Towards evidence-based physiotherapy – research challenges and needs. J Physiother. 2013;59(3):143–9. 10.1016/S1836-9553(13)70178-4. 23896329 10.1016/S1836-9553(13)70178-4

[18] Palinkas LA, Aarons GA, Horwitz S, Chamberlain P, Hurlburt M, Landsverk J. Mixed method designs in implementation research. Adm Policy Ment Health. 2011;38(1):44–53. 10.1007/s10488-010-0314-z. 20967495 10.1007/s10488-010-0314-z PMC3025112

[19] Greenhalgh T, Robert G, Macfarlane F, Bate P, Kyriakidou O. Diffusion of innovations in service organizations: systematic review and recommendations. Milbank Q. 2004;82(4):581–629. 10.1111/j.0887-378X.2004.00325.x. 15595944 10.1111/j.0887-378X.2004.00325.x PMC2690184

[20] Sims D, Cilliers F. Qualitatively speaking: deciding how much data and analysis is enough. Afr J Health Prof Educ. 2023;15(1):2–3. 10.7196/AJHPE.2023.v15i1.1657.

[21] Kiekens A, Dierckx de Casterlé B, Vandamme A-M. Qualitative systems mapping for complex public health problems: a practical guide. PLoS One. 2022;17(2):e0264463. 10.1371/journal.pone.0264463 10.1371/journal.pone.0264463 PMC8880853 35213648

[22] Luke DA, Powell BJ, Paniagua-Avila A. Bridges and mechanisms: integrating systems science thinking into implementation research. Annu Rev Public Health. 2024;45:289–306. 10.1146/annurev-publhealth-060922-040205. 10.1146/annurev-publhealth-060922-040205 38100647

[23] Pawson R, Tilley N. Realistic evaluation. London: Sage; 1997.

[24] Bach-Mortensen AM, Verboom B. Barriers and facilitators systematic reviews in health: a methodological review and recommendations for reviewers. Res Synth Methods. 2020;11(6):743–59. 10.1002/jrsm.1447. 32845574 10.1002/jrsm.1447

[25] Inoue S, Tateishi S, Harada A, Oginosawa Y, Abe H, Saeki S, et al. Qualitative study of barriers and facilitators encountered by individuals with physical diseases in returning and continuing to work. BMC Health Serv Res. 2022;22(1):1229. 10.1186/s12913-022-08604-z. 36192749 10.1186/s12913-022-08604-z PMC9531482

[26] Weiner BJ. A theory of organizational readiness for change. Implement Sci. 2009;4:67. 10.1186/1748-5908-4-67. 19840381 10.1186/1748-5908-4-67 PMC2770024

[27] Aarons GA, Ehrhart MG, Farahnak LR, Sklar M. Aligning leadership across systems and organizations to develop a strategic climate for evidence-based practice implementation. Annu Rev Public Health. 2014;35:255–74. 10.1146/annurev-publhealth-032013-182447. 24641560 10.1146/annurev-publhealth-032013-182447 PMC4348088

[28] Lewis CC, Fischer S, Weiner BJ, Stanick C, Kim M, Martinez RG. Outcomes for implementation science: an enhanced systematic review of instruments using evidence-based rating criteria. Implement Sci. 2015;10:155. 10.1186/s13012-015-0342-x. 26537706 10.1186/s13012-015-0342-x PMC4634818

[29] Caci L, Nyantakyi E, Blum K, Sonpar A, Schultes MT, Albers B, et al. Organizational readiness for change: a systematic review of the healthcare literature. Implement Res Pract. 2025;15:6. 10.1177/26334895251334536. 10.1177/26334895251334536 PMC12084713 40385227

[30] Kirk MA, Kelley C, Yankey N, Birken SA, Abadie B, Damschroder L. A systematic review of the use of the Consolidated Framework for Implementation Research (CFIR). Implement Sci. 2016;11(1):72. 10.1186/s13012-016-0437-z. 27189233 10.1186/s13012-016-0437-z PMC4869309

[31] Weiner BJ, Lewis CC, Stanick C, Powell BJ, Dorsey CN, Clary AS, et al. Psychometric assessment of three newly developed implementation outcome measures. Implement Sci. 2017;12(1):108. 10.1186/s13012-017-0635-3. 28851459 10.1186/s13012-017-0635-3 PMC5576104

[32] Greenhalgh T, Swinglehurst D. Studying technology use as social practice: the untapped potential of ethnography. BMC Med. 2011;9:45. 10.1186/1741-7015-9-45. 21521535 10.1186/1741-7015-9-45 PMC3108909

[33] Proctor E, Silmere H, Raghavan R, Hovmand P, Aarons G, Bunger A, et al. Outcomes for implementation research: conceptual distinctions, measurement challenges, and research agenda. Admin Policy Ment Health. 2011;38(2):65–76. 10.1007/s10488-010-0319-7. 10.1007/s10488-010-0319-7 PMC3068522 20957426

[34] Stanick CF, Halko HM, Dorsey CN, et al. Operationalizing the ‘pragmatic’ measures construct using a stakeholder feedback and a multi-method approach. BMC Health Serv Res. 2018;18:882. 10.1186/s12913-018-3709-2. 30466422 10.1186/s12913-018-3709-2 PMC6251134

[35] Bosch-Capblanch X, Liaqat S, Garner P. Improving the quality of trials reporting on implementation strategies for health systems research: a systematic review. Implement Sci. 2011;6:43. 10.1186/1748-5908-6-43. 21524292 10.1186/1748-5908-6-43 PMC3104485

[36] Lewis CC, Klasnja P, Powell BJ, Tuzzio L, Jones S, Walsh-Bailey C, et al. From classification to causality: Advancing understanding of mechanisms of change in implementation science. Front Public Health. 2018;6:136. 10.3389/fpubh.2018.00136. 29868544 10.3389/fpubh.2018.00136 PMC5949843

[37] Dul J. Necessary Condition Analysis (NCA): logic and methodology of “necessary but not sufficient” causality. Organ Res Methods. 2016;19(1):10–52. 10.1177/1094428115584005.

[38] Rohlfing I, Schneider CQ. A unifying framework for causal analysis in set-theoretic multimethod research. Sociol Methods Res. 2018;47(1):37–63. 10.1177/0049124115626170.

[39] Lewis CC, Klasnja P, Lyon AR, Powell BJ, Lengnick-Hall R, Buchanan G, et al. The mechanics of implementation strategies and measures: advancing the study of implementation mechanisms. Implement Sci Commun. 2022;3:114. 10.1186/s43058-022-00358-3. 36273224 10.1186/s43058-022-00358-3 PMC9588220

[40] Williams NJ. Multilevel mechanisms of implementation strategies in mental health: integrating theory, research, and practice. Adm Policy Ment Health. 2016;43(5):783–98. 10.1007/s10488-015-0693-2. 26474761 10.1007/s10488-015-0693-2 PMC4834058

[41] Rihoux B, Ragin CC, editors. Configurational comparative methods: Qualitative Comparative Analysis (QCA) and related techniques. Thousand Oaks, CA: Sage; 2009. 10.4135/9781452226569

[42] Valente TW. Social networks and health: models, methods, and applications. New York: Oxford University Press; 2010. 10.1093/acprof:oso/9780195301014.001.0001.

[43] Miller RL, Page SE. Complex adaptive systems: an introduction to computational models of social life. Princeton, NJ: Princeton University Press; 2007.

[44] Pope C, Halford S, Turnbull J, Prichard J, Calestani M, May C. Using computer decision support systems in NHS emergency and urgent care: ethnographic study using normalisation process theory. BMC Health Serv Res. 2013;13:111. 10.1186/1472-6963-13-111. 23522021 10.1186/1472-6963-13-111 PMC3614561

[45] Yin RK. Case study research and applications: design and methods. 6th ed. Thousand Oaks, CA: Sage; 2018.

[46] Stake RE. Multiple case study analysis. New York: Guilford Press; 2006.

[47] Kitson A, Harvey G, McCormack B. Enabling the implementation of evidence based practice: a conceptual framework. Qual Healthc. 1998;7(3):149–58. 10.1136/qshc.7.3.149. 10.1136/qshc.7.3.149 PMC2483604 10185141

[48] Aarons GA, Hurlburt M, Horwitz SM. Advancing a conceptual model of evidence-based practice implementation in public service sectors. Admin Policy Ment Health. 2011;38(1):4–23. 10.1007/s10488-010-0327-7. 10.1007/s10488-010-0327-7 PMC3025110 21197565

[49] Ferlie E, Fitzgerald L, Wood M, Hawkins C. The nonspread of innovations: the mediating role of professionals. Acad Manage J. 2005;48(1):117–34. 10.5465/amj.2005.15993150.

[50] Nilsen P, Potthoff S, Birken SA. Conceptualising four categories of behaviours: implications for implementation strategies to achieve behaviour change. Front Health Serv. 2022;1:795144. 10.3389/frhs.2021.795144. 36926485 10.3389/frhs.2021.795144 PMC10012728

[51] Pettigrew AM, Woodman RW, Cameron KS. Studying organizational change and development: challenges for future research. Acad Manage J. 2001;44(4):697–713. 10.5465/3069411.

[52] Manzano A. The craft of interviewing in realist evaluation. Evaluation. 2016;22(3):342–60. 10.1177/1356389016638615.

[53] Bernal JL, Cummins S, Gasparrini A. Interrupted time series regression for the evaluation of public health interventions: a tutorial. Int J Epidemiol. 2017;46(1):348–55. 10.1093/ije/dyw098. 27283160 10.1093/ije/dyw098 PMC5407170

[54] Cargo M, Mercer SL. The value and challenges of participatory research: strengthening its practice. Annu Rev Public Health. 2008;29:325–50. 10.1146/annurev.publhealth.29.091307.083824. 18173388 10.1146/annurev.publhealth.29.091307.083824

[55] Bradbury H, editor. The Sage handbook of action research. 3rd ed. Thousand Oaks, CA: Sage; 2015. 10.4135/9781473921290

[56] Bhattacharyya O, Reeves S, Zwarenstein M. What is implementation research? Rationale, concepts, and practices. Res Soc Work Pract. 2009;19(5):491–502. 10.1177/1049731509335528.

[57] Powell BJ, Waltz TJ, Chinman MJ, Damschroder LJ, Smith JL, Matthieu MM, et al. A refined compilation of implementation strategies: results from the Expert Recommendations for Implementing Change (ERIC) project. Implement Sci. 2015;10(1):21. 10.1186/s13012-015-0209-1. 25889199 10.1186/s13012-015-0209-1 PMC4328074

[58] Waltz TJ, Powell BJ, Fernández ME, Abadie B, Damschroder LJ. Choosing implementation strategies to address contextual barriers: diversity in recommendations and future directions. Implement Sci. 2019;14(1):42. 10.1186/s13012-019-0892-4. 31036028 10.1186/s13012-019-0892-4 PMC6489173

[59] Lewis CC, Boyd MR, Walsh-Bailey C, Lyon AR, Beidas RS, Mittman BS, et al. A systematic review of empirical studies examining mechanisms of implementation in health. Implement Sci. 2020;15(1):21. 10.1186/s13012-020-00983-3. 32299461 10.1186/s13012-020-00983-3 PMC7164241

[60] Rycroft-Malone J, Burton CR, Wilkinson J, Harvey G, McCormack B, Baker R, et al. Collective action for implementation: a realist evaluation of the implementation of facilitation within the PARIHS framework. Implement Sci. 2013;8:128. 10.1186/1748-5908-8-128. 26860631 10.1186/s13012-016-0380-z PMC4748518

